# Optimal Low-Cost MEMS INS/GNSS Integrated Georeferencing Solution for LiDAR Mobile Mapping Applications

**DOI:** 10.3390/s25247683

**Published:** 2025-12-18

**Authors:** Nasir Al-Shereiqi, Mohammed El-Diasty, Ghazi Al-Rawas

**Affiliations:** Civil and Architectural Engineering Department, College of Engineering, Sultan Qaboos University, Muscat 123, Oman

**Keywords:** MEMS IMU, LiDAR, georeferencing, maximum likelihood, WNN

## Abstract

Mobile mapping systems using LiDAR technology are becoming a reliable surveying technique to generate accurate point clouds. Mobile mapping systems integrate several advanced surveying technologies. This research investigated the development of a low-cost, accurate Microelectromechanical System (MEMS)-based INS/GNSS georeferencing system for LiDAR mobile mapping applications, enabling the generation of accurate point clouds. The challenge of using the MEMS IMU is that it is contaminated by high levels of noise and bias instability. To overcome this issue, new denoising and filtering methods were developed using a wavelet neural network (WNN) and an optimal maximum likelihood estimator (MLE) method to achieve an accurate MEMS-based INS/GNSS integration navigation solution for LiDAR mobile mapping applications. Moreover, the final accuracy of the MEMS-based INS/GNSS navigation solution was compared with the ASPRS standards for geospatial data production. It was found that the proposed WNN denoising method improved the MEMS-based INS/GNSS integration accuracy by approximately 11%, and that the optimal MLE method achieved approximately 12% higher accuracy than the forward-only navigation solution without GNSS outages. The proposed WNN denoising outperforms the current state-of-the-art Long Short-Term Memory (LSTM)–Recurrent Neural Network (RNN), or LSTM-RNN, denoising model. Additionally, it was found that, depending on the sensor–object distance, the accuracy of the optimal MLE-based MEMS INS/GNSS navigation solution with WNN denoising ranged from 1 to 3 cm for ground mapping and from 1 to 9 cm for building mapping, which can fulfill the ASPRS standards of classes 1 to 3 and classes 1 to 9 for ground and building mapping cases, respectively.

## 1. Introduction

Mobile mapping systems are increasingly used to generate surface elevation models. Mobile mapping systems integrate several advanced surveying technologies. The basic components of these systems include a georeferencing direct element along with a Light Detection and Ranging (LiDAR) scanner and/or a digital imager as the remote sensing component. Direct georeferencing is the determination of time-variable position and attitude (orientation) parameters for a mobile mapping system. The most common technologies used for this purpose today are integrated navigation systems composed of IMU sensors and GNSS receivers. Although each IMU and GNSS technology can, in principle, determine both position and orientation, they are typically integrated so that the GNSS receiver serves as the primary position sensor, while the IMU serves as the primary orientation sensor. IMUs are generally classified into strategic, navigation, tactical, and low-cost industrial grades based on the embedded technologies used to develop these sensors. Only navigation-grade and tactical-grade IMUs (prices ranging within tens of thousands of dollars) have been implemented in the georeferencing components of LiDAR mobile mapping systems.

Direct georeferencing of LiDAR data requires instantaneous position and attitude information for each range measurement. It should be noted that the development of the LiDAR mobile mapping system would not have been possible without direct georeferencing using a GNSS/INS integrated system. The INS algorithm primarily relies on data from the IMU gyroscopes and accelerometers. The current state-of-the-art georeferencing system for mobile mapping applications is a tactical-grade INS/GNSS integrated system. This research investigates the use of a low-cost MEMS-based INS/GNSS integrated system. The main issue with MEMS IMU gyroscopes and accelerometers is their high noise levels and time-varying, nonlinear bias instability [1]. These noise and bias instability errors require both a denoising/filtering method to suppress noise and a modeling method to rigorously remove nonlinear bias instability (time-varying and spatially correlated error) [1].

The current state-of-the-art methods for denoising MEMS IMU data include low-pass filtering [2], wavelet-based multiresolution analysis [3,4], and neural network methods [5,6,7]. However, a more accurate denoising method is required for mobile mapping applications that demand high accuracy. A highly nonlinear WNN model is proposed herein to suppress noise in MEMS-based IMU gyroscope and accelerometer data during preprocessing. Recent research highlights the critical role of precise noise and bias characterization in MEMS IMUs for improving the accuracy of geospatial measurements. Navigation solution performance is significantly affected by the high noise and bias instability inherent in MEMS gyroscopes, compared with accelerometers [6,7]. Researchers have analyzed the noise behavior and long-term bias instability of sensors [8,9]. Deep learning techniques such as Long Short-Term Memory (LSTM), autoencoders, Mixture of Experts (MoE), and Convolutional Neural Network (CNN) models have become valuable for addressing noise and bias in MEMS IMU modeling from multiple perspectives [10,11,12,13]. LSTM outperforms the other deep learning methods and is used herein in comparison with the proposed method. Also, a hierarchical learning model based on neural ordinary differential equations was introduced to represent the continuous evolution of bias [14]. However, MEMS noise and bias modeling remains an open area of research, and there is a need to develop accurate denoising and modeling techniques that preserve the stochastic characteristics of MEMS gyroscope and accelerometer measurements.

On the other hand, the current state-of-the-art method for reducing systematic bias instability error employs a classical laboratory calibration method [15,16]. However, for MEMS IMU sensors, the bias stability is inherently nonlinear, and the classical laboratory calibration method cannot accurately remove the resulting nonlinear bias instability. Hence, the remaining (residual) nonlinear bias instability errors persist in MEMS-based IMU data after implementation of the classical calibration method, which induces an artifact error into the digital elevation model produced by the mobile mapping system. Extended Kalman filters, unscented Kalman filters, and particle filters have been used to model bias instabilities in INS/GNSS integration. The three filters provide similar navigation solutions, model bias instabilities, and provide accurate navigation with GNSS outages [17]. During GNSS outages, navigation solutions drift exponentially due to residual unmodeled bias instability errors [18]. Therefore, smoothing algorithms were proposed to reduce the impact of bias instability on the navigation solution. An intelligent strategy that integrates an ANN with a conventional Rauch–Tung–Striebel (RTS) smoother was introduced to improve overall accuracy in a MEMS INS/GNSS integrated system in post-processing mode. By combining the MEMS INS/GNSS system with the suggested ANN-RTS smoother, a more cost-effective yet reasonably accurate scheme for determining position and orientation can be obtained [19,20]. Most recently, an adaptive unscented Kalman filter was investigated to improve INS/GNSS integration [21,22]. Additionally, a robust adaptive extended Kalman Filter, based on an enhanced measurement-noise covariance matrix, was investigated for monitoring and isolating abnormal disturbances in MEMS INS/GNSS vehicle navigation to improve the navigation solution [23].

However, developing an accurate denoising method and an optimal, rigorous estimator for low-cost MEM-based INS/GNSS navigation solutions remains a challenge and is the subject of extensive investigation. The main objective of this research is to develop a low-cost MEMS-based INS/GNSS navigation solution for mobile mapping applications using high-end, low-cost MEMS IMUs (prices within a few thousand dollars). A wavelet neural network (WNN) model is proposed to reduce noise, and an optimal MLE method is developed to mitigate bias instability. The developed MEMS-based INS/GNSS system is compared with the current tactical-grade-based INS/GNSS system. The accuracy of the proposed MEMS-based INS/GNSS georeferencing system is investigated to determine whether it meets the American Society for Photogrammetry and Remote Sensing (ASPRS) horizontal and vertical accuracy standards for geospatial data produced by mobile mapping applications using a LiDAR system [24].

## 2. Materials and Methods

**Figure 1:**
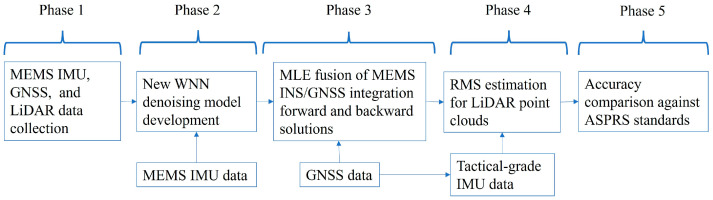
Research methodology.

The research methodology is classified into five phases as shown in Figure 1. In the first phase, data are collected using a MEMS IMU and a tactical-grade IMU, along with GNSS and LiDAR data. In the second phase, MEMS IMU gyroscope and accelerometer data are denoised using a wavelet neural network (WNN). In the third phase, a MEMS-based INS/GNSS navigation solution (positions, velocities, and attitudes) is achieved using optimal MLE hybridization for forward and backward INS/GNSS integration to reduce time-varying and spatially correlated bias instability in the MEMS-based IMU. In the fourth phase, the RMS accuracy of the proposed method is estimated and analyzed by comparing the error differences between the MEMS-based INS/GNSS integration navigation solution and the tactical-grade INS/GNSS navigation solution. In the fifth phase, the accuracy of the developed MEMS-based INS/GNSS navigation solution is evaluated to determine whether it meets the ASPRS horizontal and vertical accuracy standards for geospatial data produced using a LiDAR mobile mapping system.

### 2.1. Wavelet Neural Network (WNN) Denoising

**Figure 2:**
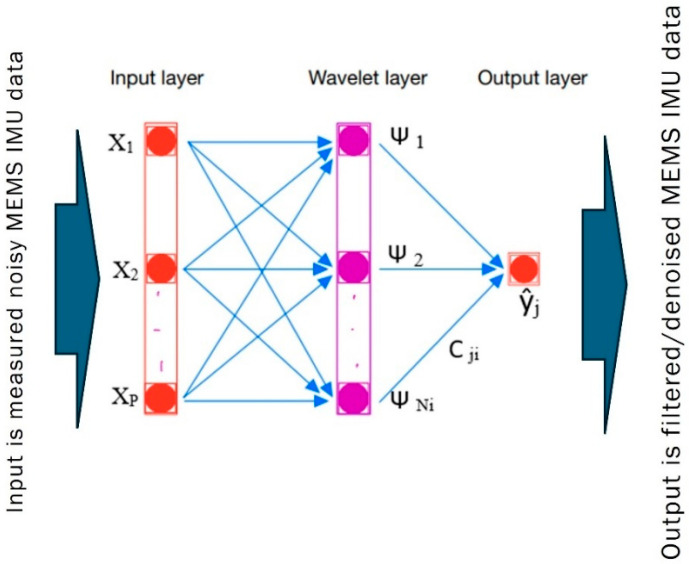
WNN denoising model architecture.

A new wavelet neural network (WNN) denoising technique is proposed to reduce noise in MEMS IMU gyroscope and accelerometer data. Figure 2 shows the proposed WNN denoising model architecture, comprising three layers: input, hidden wavelet neurons, and output [25]. The first layer serves as the input layer; the second, middle layer comprises hidden wavelet neurons; and the third layer functions as the output layer.

In this research, a model for reducing MEMS IMU noise errors is constructed utilizing a wavelet network approach, where the output ${\hat{y}}_{j}$is calculated as

(1) $${\hat{y}}_{j}=\sum_{m=1}^{Nm}c_{i\;}\Psi \;\left(a_{m\;}\left(X_{m\;}^{K}-b_{m\;}\right)\right)+w$$

where $x_{m\;}^{K\;}$ represents the input neuron, $c_{i}$ denotes weight (coefficient) variables, $a_{m}\;$represents dilation variables, $b_{m}$ stands for translation variables, w is model noise, and Ψ denotes a wavelet activation function that takes the form of a Mexican hat function of order *p*:

(2) $$\Psi \left(x\right)\;=\;(\left||x^{2}|\right|-p)\;e_{\;\;2}^{-{\left|x\right|}^{2}}$$

The WNN model weight $C_{i\;}$vector is estimated using the Levenberg–Marquardt (LM) least-squares search algorithm as follows [26]:

(3) $$C_{k+1}=C_{k}-{\left(J^{T}J+\mu I\right)}^{-1}J^{T}E$$

where J is the Jacobian matrix of the performance criteria to be minimized, μ is a learning rate value that controls the learning process, and E is the residual error vector that represents the difference between the model output vector (${\hat{Y}}_{j}$) and the desired output vector ($Y_{d})$. In practice, the dataset is divided into three subsets: the training (70%) subset to tune the model weights, the validation (15%) subset to prevent overfitting, and the unseen testing (15%) subset to evaluate the WNN model’s performance [25]. The advantage of the proposed WNN model lies in its highly nonlinear, dynamic, and flexible characteristics, including dilation and translation variables that effectively reduce noise in the nonlinear measurements from gyroscopes and accelerometers.

To reduce noise in MEMS-based IMU measurements, a WNN is implemented using windows of varying sizes to evaluate the performance of different WNN architectures. The input layer includes individual MEMS-IMU data from three gyroscope measurements and three accelerometer measurements with predefined window sizes (10, 15, 20, and 25), the hidden layer includes a wavelet function (Mexican hat) as an activation function, and the output layer contains one desired output for three gyroscope measurements and three accelerometer measurements.

### 2.2. Maximum Likelihood Estimator (MLE) Method

An optimal MLE method that integrates forward and backward MEMS-based INS/GNSS solutions is proposed to reduce MEMS IMU bias instability and thereby improve the final MEMS-based INS/GNSS navigation solution. The optimal MLE method is considered a rigorous approach that can be employed with navigation solutions to provide a hybrid, optimal solution estimate and associated uncertainties. Assume that there is a number (*N*) of MEMS-based INS/GNSS navigation solutions (${\hat{X}}_{1}$ to ${\hat{X}}_{N}$) at all epochs and associated covariance matrices ($S_{{\hat{X}}_{1}{\hat{X}}_{1}}$ to $S_{{\hat{X}}_{N}{\hat{X}}_{N}}$) using an extended Kalman filter approach. Then, the hybrid optimal MEMS-based INS/GNSS navigation solution (${\hat{X}}_{h}$) and associated covariance matrix ($S_{{\hat{X}}_{h}{\hat{X}}_{h}}$) can be estimated using the optimal MLE method. The optimal MLE method is developed based on the maximization of the likelihood function [27,28]:

(4) $$L(X)=\prod_{i=1}^{N}P(X_{i})$$

(5) $$P\left(X_{i}\right)={\left(2\pi \right)}^{-d/2}{\left|S_{{\hat{X}}_{i}{\hat{X}}_{i}}\right|}^{-1/2}\exp(-\frac{1}{2}(X_{i}-{\hat{X}}_{i}{)}^{T}{S_{{\hat{X}}_{i}{\hat{X}}_{i}}}^{-1}(X_{i}-{\hat{X}}_{i}))$$

where L(X) is the likelihood function, *N* is the number of models, and $P(X_{i})$ is the multivariate probability density function for a single model with *d* variate values with multivariate normal distribution $MVN({\hat{X}}_{i},S_{{\hat{X}}_{i}{\hat{X}}_{i}})$ [27].

The objective is to estimate the hybrid optimal MEMS-based INS/GNSS navigation solution (${\hat{X}}_{H}$) that maximizes the likelihood function and guarantees the highest likelihood estimate for the hybrid optimal navigation solution using a rigorous solution (optimal estimation). To simplify the analysis, the natural logarithm of the likelihood function is taken, and the log-likelihood l(X) is used; hence, it can be simplified as the summation of the multivariate probability density functions as follows [28,29]:

(6) $$l(X)=ln\left(\prod_{i=1}^{N}P(X_{i})\right)=\;\sum_{i=1}^{N}ln(P(X_{i}))$$

To estimate the hybrid optimal MEMS-based INS/GNSS navigation solution (${\hat{X}}_{H}$), the expectation of the first partial derivative of the log-likelihood function at the MEMS-based INS/GNSS navigation solution at $X={\hat{X}}_{H}$ shall equal zero as follows [28,29]:

(7) $${E\left(\frac{\partial (l(X))}{\partial X}\right)\rfloor }_{X={\hat{X}}_{H}}=0$$

The simplification of Equation (7) provides an estimation of the hybrid optimal MEMS-based INS/GNSS navigation solution (${\hat{X}}_{H}$) using the following formula [28]:

(8) $${\hat{X}}_{H}={\left(\sum_{i=1}^{N}{S_{{\hat{X}}_{i}{\hat{X}}_{i}}}^{-1}\right)}^{-1}.\;\sum_{i=1}^{N}{S_{{\hat{X}}_{i}{\hat{X}}_{i}}}^{-1}{\hat{X}}_{i}$$

The covariance matrix ($S_{{\hat{X}}_{H}{\hat{X}}_{H}}$) of the hybrid MEMS-based INS/GNSS navigation solution uses the negative of the expectation of the inverse Hessian matrix, which represents the second partial derivative of the log-likelihood function at a MEMS-based INS/GNSS navigation solution value at $X={\hat{X}}_{H}$, and is represented as follows [29,30]:

(9) $$S_{{\hat{X}}_{H}{\hat{X}}_{H}}={\left(-{E(\frac{\partial^{2}(l(X))}{\partial X^{2}})|}_{X={\hat{X}}_{H}}\right)}^{-1}$$

Then, Equation 9 is simplified to provide an estimation of the covariance function ($S_{{\hat{X}}_{h}{\hat{X}}_{h}}$) using the following equation [28]:

(10) $$S_{{\hat{X}}_{H}{\hat{X}}_{H}}={\left(\sum_{i=1}^{N}{S_{{\hat{X}}_{i}{\hat{X}}_{i}}}^{-1}\right)}^{-1}$$

Equations (8) and (10) are employed to estimate the hybrid optimal MEMS-based INS/GNSS navigation solution (${\hat{X}}_{H}$) and associated covariance matrix ($S_{{\hat{X}}_{H}{\hat{X}}_{H}}$), respectively, using the optimal MLE method with two forward and backward solutions, where *N* = 2, ${\hat{X}}_{1}$ and $S_{{\hat{X}}_{1}{\hat{X}}_{1}}$ are obtained from a forward MEMS-based INS/GNSS navigation solution, and ${\hat{X}}_{2}$ and $S_{{\hat{X}}_{2}{\hat{X}}_{2}}$ are obtained from the backward MEMS-based INS/GNSS navigation solution. The MLE methodology for hybridizing forward and backward navigation solutions from MEMS-based INS/GNSS integration is considered optimal because it maximizes the likelihood of the navigation solutions, minimizes estimation variance, handles system nonlinearities, and adapts the solution to remove residual MEMS instability errors.

### 2.3. RMS Error Estimation of Georeferenced LiDAR Point Clouds

**Figure 3:**
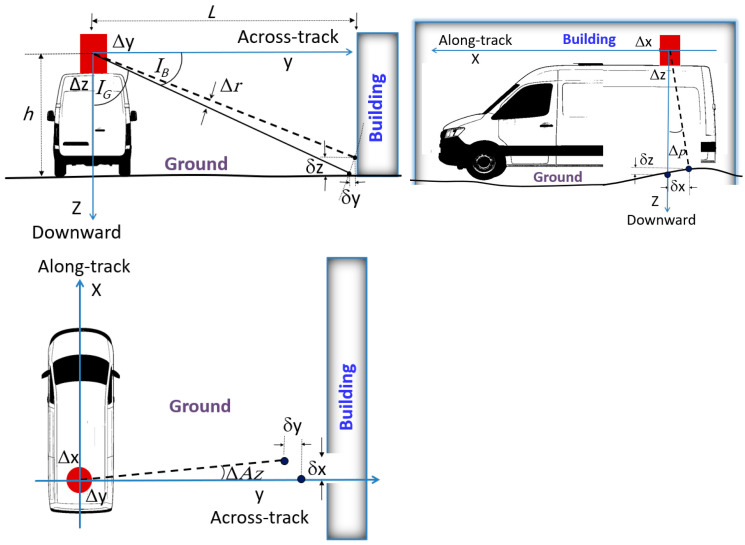
Ground and building point cloud root-mean-square errors.

Accuracy can vary significantly depending on the environment. Thus, the results are analyzed for different ground and building cases, as shown in Figure 3. The figure shows three views, the front, side, and plan views, of a mobile mapping vehicle with estimated RMSE position errors (Δx, Δy, and Δz) and RMSE attitude errors (Δ*r*, Δ*p*, and Δ*Az*) of the MEMS-based INS/GNSS georeferencing system that can be utilized to obtain the RMSE of point clouds. For the ground case, the height *h* equals 2.5 m, and *I_G_* is the laser incidence angle, which equals 45 degrees. For the building case, the distance from the scanner to the building is *L* and varies (5, 10, 15, 20, 30, 35, 40, 45, 50, or 55 m), whereas *I_B_* is the incidence angle and equals 45°.

From the geometrical characteristics of Figure 3, the RMSE position errors (Δx, Δy, and Δz) and RMSE attitude errors (Δ*r*, Δ*p*, and Δ*Az*) of the MEMS-based INS/GNSS georeferencing system produce point cloud RMSE errors (δx, δy, and δz), and from these errors, the RMSE of the ground point cloud in the horizontal direction, the vertical direction, and 3D are estimated. Similarly, the RMSE of the building point cloud is estimated in the horizontal and vertical directions and in three dimensions. To evaluate the LiDAR point cloud horizontal accuracy, vertical accuracy, and 3D accuracy in each case (ground and building), the following equations are derived:

1. Vertical RMS error for the ground case:

(11) $${RMSE}_{V}^{G}=\sqrt{∆z^{2\;}+{\left(\frac{h}{cos(I_{G})}\tan\left(∆r\right)\sin\left(I_{G}+∆r\right)\right)}^{2}+{\left(h\tan\left(∆p\right)\sin\left(∆p\right)\right)}^{2}}$$

2. Horizontal RMS error for the ground case:

(12) $${RMSE}_{H}^{G}=\sqrt{\begin{array}{c} {∆x}^{2}+{∆y}^{2}+{\left(\frac{h}{\cos\left(I_{G}\right)}tan\left(∆r\right)cos\left(I_{G}+∆r\right)\right)}^{2}+{\left(h\tan\left(∆p\right)\cos\left(∆p\right)\right)}^{2}\\ +{\left(h\tan\left(∆Az\right)cos(∆Az)\right)}^{2}+{\left(h\tan\left(∆Az\right)sin(∆Az)\right)}^{2\;} \end{array}}$$

3. Three-dimensional RMS error for the ground case:

(13) $${RMSE}_{3D}^{G}=\sqrt{{\left({RMSE}_{H}^{G}\right)}^{2}+{\left({RMSE}_{V}^{G}\right)}^{2}}$$

4. Vertical RMS error for the building case:

(14) $${RMSE}_{V}^{B}=\sqrt{∆y^{2\;}+{\left(\;\frac{L}{cos{(I}_{B})}\tan\left(∆r\right)sin(I_{B}+∆r)\right)}^{2}+{\left(L\tan\left(∆Az\right)sin(∆Az)\right)}^{2}}$$

5. Horizontal RMS error for the building case:

(15) $${RMSE}_{H}^{B}=\sqrt{\begin{array}{c} {∆x}^{2}+{∆z}^{2}+{\left(\frac{L}{\cos\left(I_{B}\right)}tan\left(∆r\right)cos\left(I_{B}+∆r\right)\right)}^{2}+{\left(L\tan\left(∆Az\right)\cos\left(∆Az\right)\right)}^{2}\\ +{\left(L\tan\left(∆p\right)cos(∆p)\right)}^{2}+{\left(L\tan\left(∆p\right)sin(∆p)\right)}^{2} \end{array}}$$

6. Three-dimensional RMS error for the building case:

(16) $${RMSE}_{3D}^{B}=\sqrt{{\left({RMSE}_{H}^{B}\right)}^{2}+{\left({RMSE}_{V}^{B}\right)}^{2}}$$

where *h* is the scanner height above the ground (ground mapping case), *L* is the perpendicular distance from scanner to building (ground mapping case), *I_G_* is the laser incidence angle to the ground surface, *I_B_* is the laser incidence angle to the building, Δx is the position RMSE in the X direction, Δy is the position RMSE in the Y direction, Δz is the position RMSE in the Z direction, Δ*r* is the roll RMSE from the INS, Δ*p* is the pitch RMSE from the INS, and Δ*Az* is the azimuth RMSE from the proposed MEMS-based INS/GNSS navigation solution.

### 2.4. American Society of Photogrammetry and Remote Sensing (ASPRS) Standard

The LiDAR point cloud accuracies are classified based on the estimated RMSE for ground and building cases (${RMSE}^{G}\;and\;{RMSE}^{B})$ and using ASPRS standards. These classes (-cm) are classified by RMSE value (≤#) to ensure that the point clouds produced in geospatial data across various applications (ground and building cases) are categorized according to the ASPRS standard classes shown in Table 1 [24]. This classification reflects the suitability of the processed data for specific applications, based on the observed accuracy and reliability metrics from previous phases.

## 3. Test Description and Data Sets

**Figure 4:**
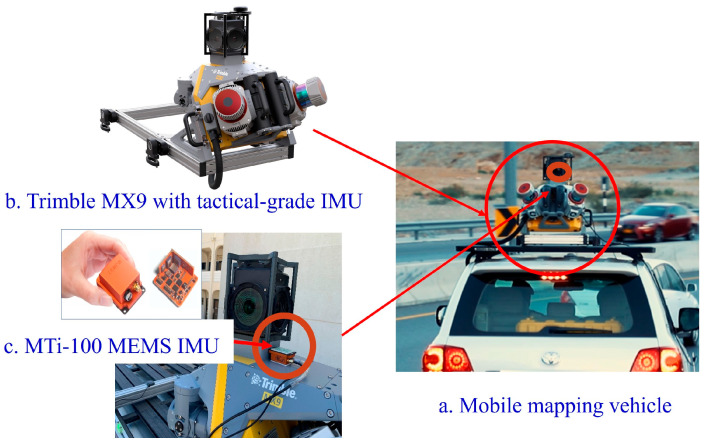
Trimble MX9 Mobile Mapping System [31] and MTi-100 MEMS-grade IMU [32] along with RTK GNSS system.

**Figure 5:**
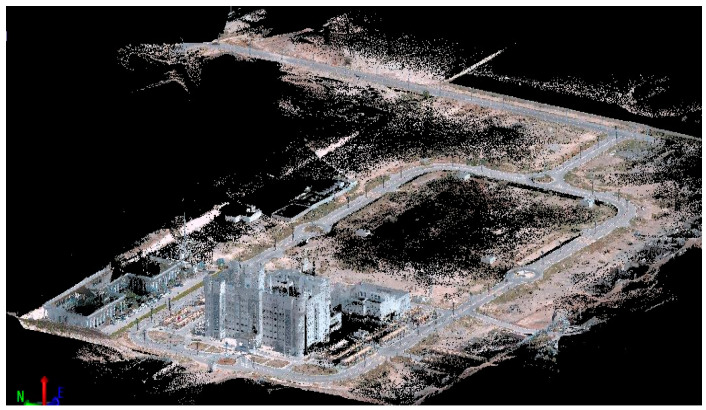
Example of point cloud data from laser scan data (ground and building cases).

**Figure 6:**
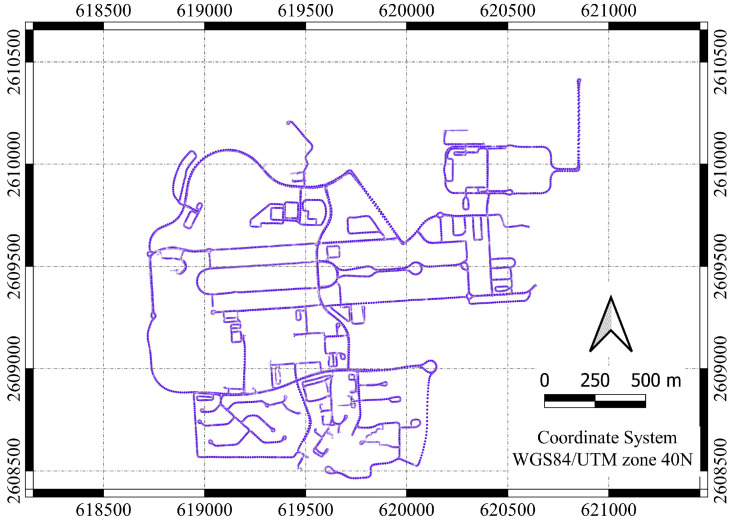
Mobile mapping test trajectory.

Data from three gyroscopes (angular rate of change) and three accelerometers (acceleration) from both the Trimble MX9 mobile mapping system with a tactical-grade IMU [31] and the Xsend MTi-100 MEMS-grade IMU [32], along with accurate GNSS positions, were collected on the SQU campus on 29 March 2022, as illustrated in Figure 4. The MTi-100 MEMS-grade IMU was located on the top of the Trimble MX9 system. Table 2 shows the specifications of the MTi-100 MEMS-grade IMU. An example of experimental point cloud data derived from laser scanning (ground and building) is shown in Figure 5. The test trajectory is shown in Figure 6.

## 4. Results and Discussion

**Figure 7:**
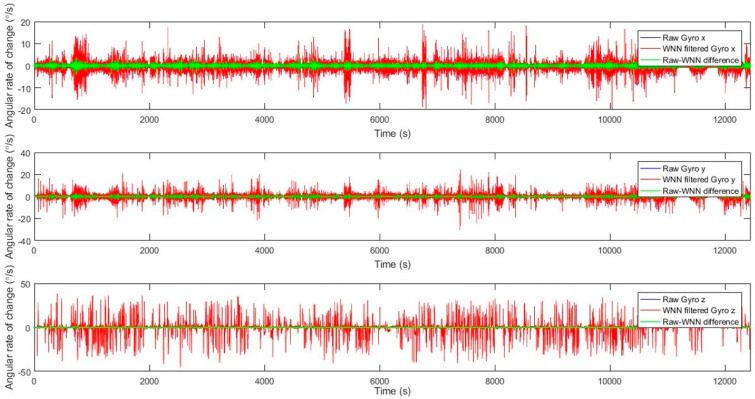
Raw, WNN filtered, and difference results for MEMS angular rate of change in x, y, and z directions with window size of 20 inputs.

**Figure 8:**
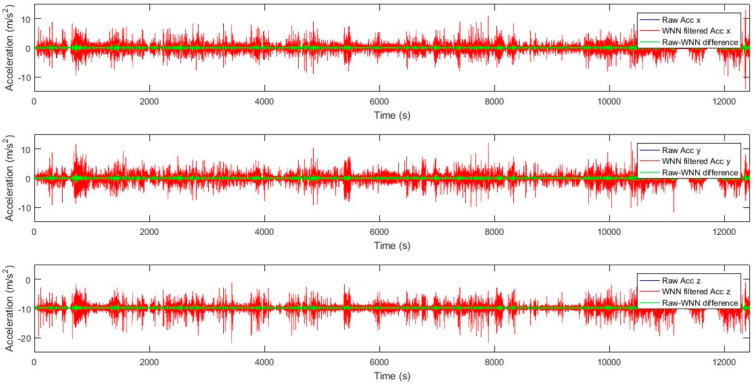
Raw, WNN filtered, and difference results for MEMS acceleration in x, y, and z directions with a window size of 20 inputs.

The MEMS IMU datasets of three accelerometer measurements (Acc x, Acc y, and Acc z) and three gyroscope measurements (Gyro x, Gyro y, and Gyro z) that were contaminated by high levels of noise and bias instability errors were processed using a WNN model to produce filtered/denoised MEMS-based data for these six components. WNN attenuates noise components while learning to recognize and maintain real motion patterns. WNN models with window sizes of 10, 15, 20, and 25 were trained in MATLAB 2019, and all models employed the Levenberg–Marquardt algorithm for supervised learning. Table 3 presents an example of WNN modeling results for a window size of 20 inputs, yielding a superior solution; the optimal WNN structure is listed along with the model mean-squared error (MSE), model correlation, and number of estimated model parameters. These optimal WNN models were achieved at the lowest model MSE and the highest model correlation. The model MSE for MEMS-based gyroscopes ranged from 0.1231 to 0.0241, with model correlation ranging from 74% to 94%; the model MSE for MEMS-based accelerometers ranged from 0.0116 to 0.0064, with model correlation ranging from 87% to 89%. Figure 7 shows raw, denoised, and difference (noise) results for the MEMS gyroscopes’ angular rate of change data measured in the x, y, and z directions from a WNN with a window size of 20 inputs. It is shown that WNN denoising for the x and y axes of the gyroscope yields noise reductions of 14% to 20%, whereas the z axis shows lower noise reductions of 1.5% to 2%. Figure 8 shows raw, denoised, and difference (noise) results for the MEMS gyroscopes’ angular rate of change data measured in the x, y, and z directions from a WNN with a window size of 20 inputs. It is shown that WNN denoising for the accelerometers’ x, y, and z axes yields noise reductions in the range of 9% to 11%.

**Figure 9:**
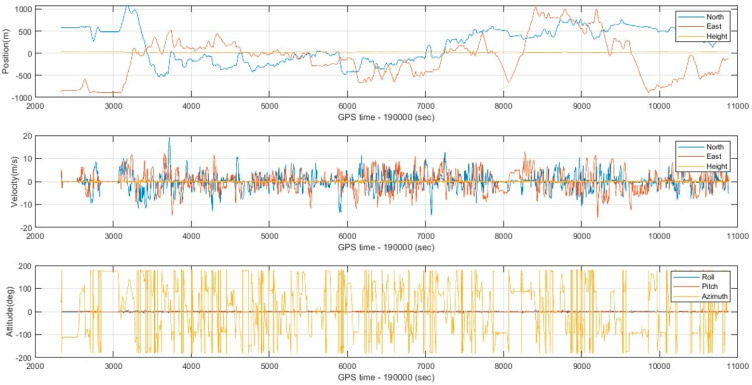
Position, velocity, and attitude solutions from the optimal MLE-based MEMS-INS/GNSS solution with a WNN denoising window size of 20 inputs.

**Figure 10:**
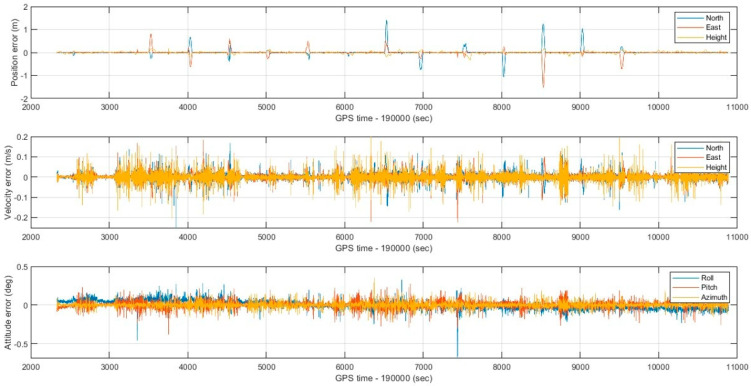
Position, velocity, and attitude errors with 15 artificial gaps of GNSS outages lasting 60 s and a WNN denoising window size of 20 inputs.

**Figure 11:**
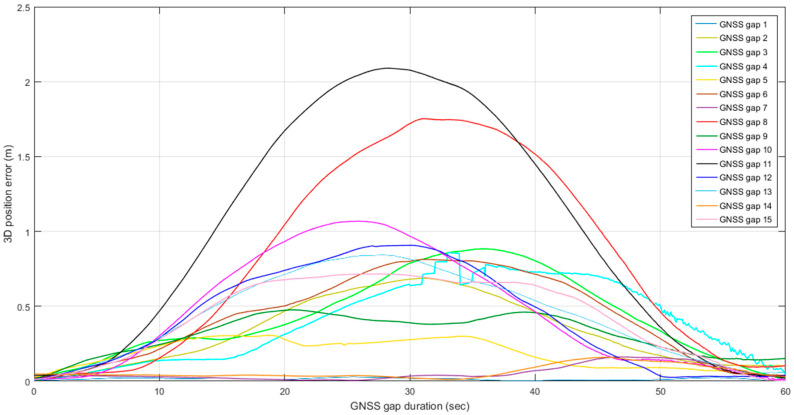
Accumulated 3D position errors with 15 artificial gaps of GNSS outages lasting 60 s and a WNN denoising window size of 20 inputs.

**Figure 12:**
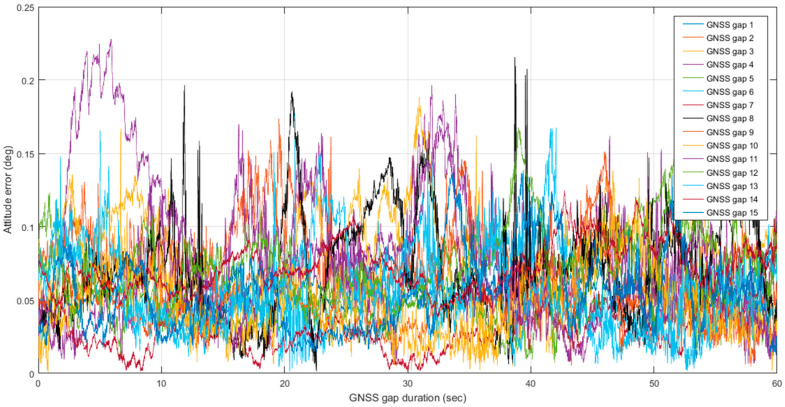
Accumulated 3D attitude errors with 15 artificial gaps of GNSS outages lasting 60 s and a WNN denoising window size of 20 inputs.

To implement the proposed optimal MLE method, the WNN-based denoised MEMS gyroscopes and accelerometers are integrated with an RTK GNSS position solution using an extended Kalman filter to estimate forward and backward MEMS INS/GNSS integrated navigation solutions. Then, the optimal MLE method is implemented using forward and backward navigation solutions to estimate the hybrid MEMS INS/GNSS integration solution, enhanced by WNN denoising. The optimal MLE method is implemented to reduce the effects of residual bias instabilities, which the extended Kalman filters cannot suppress completely. The performance of the optimal MLE method solution was evaluated against the Trimble MX9 reference system under GNSS outages of 60 s. Figure 9 shows the optimal MLE-based position (north, east, and height), velocity (north, east, and height), and attitude (roll, pitch, and azimuth) for the MEMS INS/GNSS navigation solution with MEMS WNN denoising using an optimal window size of 20 inputs. Figure 10 shows the position, velocity, and attitude error difference between the optimal MLE-based MEMS INS/GNSS navigation solution and the reference Trimble MX9 integration solution with 15 artificial gaps of GNSS outages of 60 s. Figure 11 and Figure 12 show the accumulated 3D position and attitude errors during the GNSS outages. Although error increases are observed due to residual instability errors, the optimal MLE method effectively reduces their impact, with maximum position errors below 2 m and attitude errors below 0.25 degrees. Table 4 shows the position and attitude RMSE estimated from the error difference between the optimal MLE-based MEMS INS/GNSS navigation solution with MEMS WNN filtering of different input window sizes and the Trimble MX9 navigation solution.

The comparative analysis of the RMSE during GNSS outages shows that the WNN model with a 20-input window configuration achieves the optimal navigation solution, with a 3D position RMSE of 0.5385 m and a 3D attitude RMSE of 0.0678 degrees. This optimal MLE-based navigation solution, with a WNN model and a 20-input window, provides an approximately 11% improvement in accuracy over solutions obtained with other window sizes. A comparison was made between the MLE navigation solution with WNN denoising using a window of 20 inputs and the MLE navigation solution with deep learning-based Long Short-Term Memory (LSTM)–Recurrent Neural Network (RNN), namely an LSTM-RNN denoising model using a window of 20 inputs, to further test the accuracy of the proposed WNN denoising model. Table 5 shows the position and attitude RMSE of the MLE-based MEMS INS/GNSS navigation solution during GNSS outages with WNN denoising, compared with the navigation solution using a machine learning LSTM-RNN denoising model. The comparative analysis shows that the WNN model achieves a navigation solution with a 3D position RMSE of 0.5385 m and a 3D attitude RMSE of 0.0678 degrees; however, the LSTM-RNN model achieves a navigation solution with a 3D position RMSE of 0.572 m and a 3D attitude RMSE of 0.0682 degrees. The optimal MLE navigation solution using the WNN denoising model provides an approximately 11% improvement in accuracy, and the navigation solution using the LSTM-RNN denoising model provides an approximately 5.6% improvement in accuracy when both solutions are compared with the navigation solution using raw noisy measurements. Therefore, the navigation solution using the WNN denoising model outperforms the navigation solution using the LSTM-RNN denoising model.

**Figure 13:**
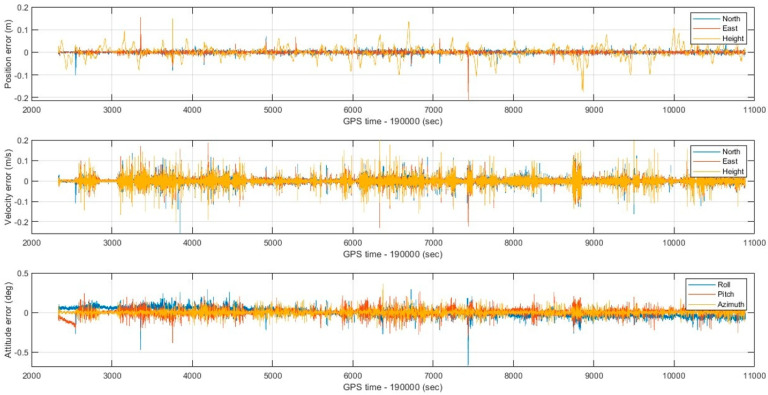
The error differences between the optimal MLE-based MEMS INS/GNS navigation solution and the reference Trimble MX9 integration solution without GNSS outages.

The accuracy of the MEMS-based INS/GNSS navigation solution (WNN denoising with a 20-input window) was evaluated using the RMSE, and the georeferencing solution was classified in accordance with the American Society for Photogrammetry and Remote Sensing (ASPRS, 2023) standards. The optimal MLE-based MEMS INS/GNS navigation solution, without GNSS outages, was tested to assess its compliance with ASPRS standards. Figure 13 shows the differences in error between the optimal MLE-based MEMS INS/GNS navigation solution and the reference Trimble MX9 integration solution. Table 6 presents a comparative analysis (based on RMSE values) between the forward-only navigation solution and the optimal MLE-based navigation solution using WNN denoising with a 20-input window. It is shown that the optimal MLE-based navigation solution outperforms the forward-only navigation solution by about 12% during GNSS outages.

**Figure 14:**
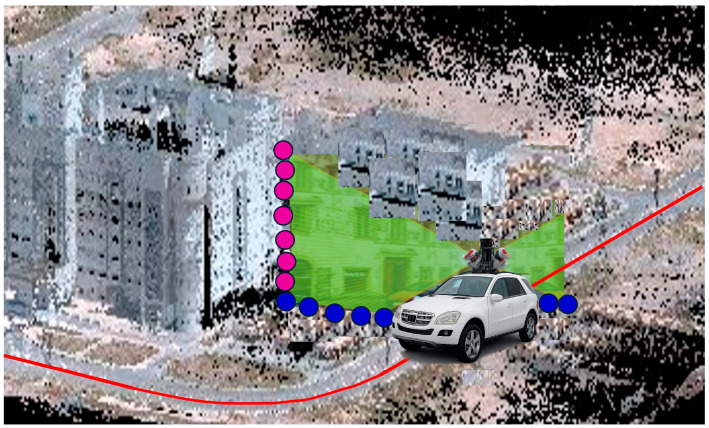
Schematic plot showing the trajectory (red line) for the LiDAR point cloud of ground (blue points) and building (purple points) cases using a dual LiDAR system.

Subsequently, the analysis was performed separately for the accuracy of the ground and building point clouds, using the RMSE values of the optimal MLE-based MEMS-INS/GNSS integrated navigation solution reported in Table 5. Figure 14 illustrates an example of a vehicle trajectory and the locations of the LiDAR point clouds. The horizontal and vertical RMSEs for ground and building cases were calculated using the formulas described in Section 2. Table 7 and Table 8 show the RMSE results for the MEMS-INS/GNSS system for both the ground and building point cloud datasets, where the RMSE values estimated for the ground case using a vehicle height of 2.5 m were 0.88 cm, 2.7 cm, and 2.8 cm for the horizontal RMSE, the vertical RMSE, and the 3D RMSE, respectively. For the building case, when distances ranging from 5 m to 55 m were used, the horizontal RMSE ranged from 2.8 cm to 7.1 cm, the vertical RMSE ranged from 0.7 cm to 4.6 cm, and the 3D RMSE ranged from 2.9 cm to 8.5 cm. To assess the geospatial quality of the produced data, the results were compared against the ASPRS accuracy classification standards. The ASPRS standards categorize data into classes based on RMSE thresholds for horizontal, vertical, and 3D positional accuracy. The classification results are summarized in Table 9 and Table 10. For the ground mapping case, the accuracy of point clouds generated by a LiDAR mobile mapping system and georeferenced using a MEMS-based INS/GNSS integration solution met class 1 in horizontal accuracy, class 3 in vertical accuracy, and class 3 overall for 3D accuracy. In the building mapping case, the accuracy of point clouds generated by a LiDAR mobile mapping system and georeferenced using a MEMS-based INS/GNSS integration solution ranged from class 3 to class 9, with the highest accuracy achieved at shorter sensor-to-building distances.

## 5. Conclusions

The development of a low-cost MEMS-based INS/GNSS navigation system for mobile mapping applications was investigated using low-cost MEMS IMU sensors. A WNN model was proposed to reduce noise, and an optimal MLE method was developed to reduce bias and instability errors. The developed MEMS-based INS/GNSS system was compared with the current tactical-grade-based INS/GNSS system. The accuracy of the proposed MEMS-based INS/GNSS georeferencing system was investigated to determine whether it meets the ASPRS horizontal and vertical accuracy standards for geospatial data produced by LiDAR mobile mapping systems. It was found that the proposed WNN denoising method improved the MEMS-based INS/GNSS integrated navigation solution accuracy by approximately 11%, and the optimal MLE method outperformed the forward-only navigation solution accuracy by approximately 12% without GNSS outages. The proposed WNN denoising outperformed the current state-of-the-art LSTM-RNN denoising model. Additionally, it was found that, depending on the sensor–object distance, the accuracy of LIDAR georeferenced point clouds obtained using the proposed optimal MLE-based MEMS INS/GNSS integrated navigation solution with WNN denoising ranged from 1 to 3 cm for ground mapping applications and from 1 to 9 cm for building mapping applications. The achieved point cloud accuracy meets the ASPRS standards for classes 1 to 3 and 1 to 9 for ground and building mapping, respectively. These findings support the feasibility of MEMS-based INS/GNSS integrated with the proposed WNN denoising model and the optimal MLE method as an efficient direct georeferencing system for LiDAR mobile mapping applications. Therefore, it is recommended to use the WNN denoising model and the optimal MLE method, along with a MEMS INS/GNSS integrated navigation solution, for georeferencing LiDAR mobile mapping systems, thereby enabling them to meet a diverse range of classes according to the APRS standards.

The significant contribution is that the proposed WNN denoising model and MLE method can be utilized to provide accurate georeferencing solutions when a MEMS INS/GNSS integrated system is employed for LiDAR mobile mapping applications. Therefore, the proposed high-end, low-cost (a few thousand dollars) MEMS INS/GNSS integrated system can achieve comparable accuracy to the current state-of-the-art, costly (tens of thousands of dollars), tactical-grade INS/GNSS integration systems when noise and instability errors are accurately reduced. A limitation of the proposed WNN denoising model is the selection of the sliding window (input-layer size), for which the optimal window size can be determined only through trial and error and is specific to the MEMS IMU under investigation. For example, the assessment for the current MTi-100 MEMS IMU showed that a window of 20 inputs is recommended for this specific MEMS IMU system.

## Figures and Tables

**Table 1:** ASPRS standard classification for geosocial point cloud data [24], where # is the number sign.

Accuracy Class	RMSE
#-cm	≤#

**Table 2:** MTi-100 MEMS IMU specifications [32].

Parameter	Gyroscope	Accelerometer
Bias stability	10 deg/h	0.05 mg
Initial bias (turn-on)	<30 deg/h	<20 mg
Random walk (noise density)	3 deg/√h	0.3 m/s^2^/√h
Scale factor error (nonlinearity)	500 ppm	600 ppm

**Table 3:** An example of WNN modeling results for a window size of 20 inputs.

MEMS IMU Sensor	Optimal WNN Structure	Model MSE	Model Correlation (%)	Number of WNN Parameters
Gyro x	20-48-1	0.1231	74	1077
Gyro y	20-86-1	0.0718	82	1913
Gyro z	20-79-1	0.0241	98	1789
Acc x	20-88-1	0.0098	89	1957
Acc y	20-44-1	0.0116	89	989
Acc z	20-118-1	0.0064	87	2617

**Table 4:** Overall RMSE values for MEMS-based INS/GNSS navigation solution with 15 artificial gaps of GNSS outages lasting 60 s.

**MLE-Based RMSE**	**Raw with Noise**	**WNN Denoising Window Size**			
**Position Errors**		**10**	**15**	**20**	**25**
RMSE (Δx) (m)	0.4088	0.3929	0.434	0.3341	0.3669
RMSE (Δy) (m)	0.4424	0.3832	0.4389	0.414	0.4204
RMSE (Δz) (m)	0.067	0.0709	0.0617	0.0833	0.065
3D Position RMSE (m)	0.606	0.5534	0.6203	0.5385	0.5617
**Attitude Error**	**Raw**	**10**	**15**	**20**	**25**
RMSE (Δr) (deg)	0.0497	0.0478	0.0508	0.0481	0.0499
RMSE (Δp) (deg)	0.0271	0.0293	0.0291	0.0275	0.028
RMSE (ΔAz) (deg)	0.0392	0.0468	0.039	0.039	0.0389
3D Attitude RMSE (deg)	0.0688	0.073	0.0703	0.0678	0.0692

**Table 5:** Comparison of RMSE values for MEMS-based INS/GNSS navigation solution using WNN denoising model and LSTM-RNN denoising model with window of 20 inputs and 15 artificial gaps of GNSS outages lasting 60 s.

**MLE-Based RMSE**	**Raw with Noise**	**Model Comparison**	
**Position Errors**		**WNN Denoising**	**LSTM-RNN Denoising**
RMSE (Δx) (m)	0.4088	0.3341	0.3454
RMSE (Δy) (m)	0.4424	0.414	0.4516
RMSE (Δz) (m)	0.067	0.0833	0.0661
3D Position RMSE (m)	0.606	0.5385	0.572
**Attitude Error**	**Raw**	**WNN**	**LSTM**
RMSE (Δr) (deg)	0.0497	0.0481	0.0479
RMSE (Δp) (deg)	0.0271	0.0275	0.0284
RMSE (ΔAz) (deg)	0.0392	0.039	0.0393
3D Attitude RMSE (deg)	0.0688	0.0678	0.0682

**Table 6:** RMSE values estimated from the error differences between the forward-only navigation solution and the optimal MLE-based navigation solution.

RMSE Parameter	Forward Solution	Optimal MLE-Based Solution
RMSE (Δx) (m)	0.0055	0.0077
RMSE (Δy) (m)	0.0058	0.0067
RMSE (Δz) (m)	0.027	0.0301
3D Position RMSE (m)	0.0282	0.0319
RMSE (Δr) (deg)	0.0475	0.05
RMSE (Δp) (deg)	0.0331	0.035
RMSE (ΔAz) deg)	0.0358	0.0445
3D Attitude RMSE (deg)	0.0681	0.0755

**Table 7:** LiDAR georeferenced point cloud accuracy (RMSE) for ground mapping case.

Height (h)	Horizontal RMSE_H_ (cm)	Vertical RMSE_V_	Three Dimensions RMSE_3D_ (cm)
Gyroscope x	0.88	2.70	2.80

**Table 8:** LiDAR georeferenced point cloud accuracy (RMSE) for building mapping case.

Distance (L)	Horizontal RMSE_H_ (cm)	Vertical RMSE_V_	Three Dimensions RMSE_3D_ (cm)
5 m	2.8	0.7	2.8
10 m	3.0	1.0	3.2
15 m	3.3	1.4	3.6
20 m	3.7	1.8	4.1
25 m	4.1	2.2	4.7
30 m	4.5	2.6	5.2
35 m	5.0	3.0	5.8
40 m	5.5	3.4	6.5
45 m	6.1	3.8	7.2
50 m	6.6	4.2	7.8
55 m	7.1	4.6	8.5

**Table 9:** ASPRS classification for LiDAR georeferenced point clouds in ground mapping case.

Height (h)	Horizontal		Vertical		Three Dimensions	
	RMSE (cm)	Accuracy Class	RMSE (cm)	Accuracy Class	RMSE (cm)	Accuracy Class
2.5 m	0.88	class 1	2.70	class 3	2.80	class 3

**Table 10:** ASPRS classification for LiDAR point clouds in building mapping case.

Height (h)	Horizontal		Vertical		Three Dimensions	
	RMSE (cm)	Accuracy Class	RMSE (cm)	Accuracy Class	RMSE (cm)	Accuracy Class
5 m	2.8	class 3	0.7	class 1	2.8	class 3
10 m	3.0	class 3	1.0	class 1	3.2	class 4
15 m	3.3	class 4	1.4	class 2	3.6	class 4
20 m	3.7	class 4	1.8	class 2	4.1	class 5
25 m	4.1	class 5	2.2	class 3	4.7	class 5
30 m	4.5	class 5	2.6	class 3	5.2	class 6
35 m	5.0	class 5	3.0	class 3	5.8	class 6
40 m	5.5	class 6	3.4	class 4	6.5	class 7
45 m	6.1	class 7	3.8	class 4	7.2	class 8
50 m	6.6	class 7	4.2	class 5	7.8	class 8
55 m	7.1	class 8	4.6	class 5	8.5	class 9

## Data Availability

Data available upon request from the corresponding author.
